# Hypertension Treatment in Patients with Metabolic Syndrome and/or Type 2 Diabetes Mellitus: Analysis of the Therapy Effectivity and the Therapeutic Inertia in Outpatient Study

**DOI:** 10.1155/2018/8387613

**Published:** 2018-04-01

**Authors:** Štefan Farský, Andrea Strišková, Marián Borčin

**Affiliations:** House of the Heart (Dom Srdca), Slovak League against Hypertension, Martin, Slovakia

## Abstract

We have analysed the database of 1,595 consecutive patients visiting our department of cardiology and internal medicine clinic in 2005–2014. The analysis included 13,990 visit records, and the average number of visits per patient was 8.5 ± 7.0. Our goals were to evaluate the effectivity of hypertension treatment as for drug choice, decrease of sBP and dBP associated with a certain drug, a drug combination, and therapeutic inertia in patients with metabolic syndrome and/or diabetes mellitus. The final number of patients for analysis who fulfilled the inclusion criteria for interpenetration of both diagnostic circles was 570. *Results*. 15% of patients were treated using hypertension monotherapy, 70% of patients were treated using 2- to 4-drug combination therapy, and 15% of patients were treated using 5- to 6-drug combination. The drugs used most frequently were perindopril (perin), nitrendipine (nitre), amlodipine (amlo), telmisartan (telmi), hydrochlorothiazide (hydro), rilmenidine, and nebivolol (used in >100 patients). The most significant decrease of sBP was associated with treatment by nitre, hydro, telmi, and urapidil (>19 mmHg). The most significant decrease of dBP was associated with treatment by nitre, hydro, telmi, and verapamil (>10 mmHg). The most significant decrease of both sBP and dBP was associated with treatment using 3-drug combination of telmi + hydro + spironolactone (41 and 16 mmHg, resp.), telmi + hydro + nitre (34 and 15 mmHg, resp.), and telmi + hydro + urapidil (34 and 15 mmHg, resp.). At the last visit, 281 out of 413 patients at the first visit had sBP >140 mmHg (68%); that is, sBP control was 32%. At the last visit, 76 patients out of 217 at the first visit had dBP >90 mmHg (35%); that is, dBP control was 65%. Therapeutic inertia was calculated by evaluating the proportion of visits at which sBP was above the target for eligible visits minus the proportion of visits where the change was made in antihypertensive treatment (AHT), either medication type or dose, over the number of eligible visits, with the resultant value multiplied by the mean of the difference between the actual sBP and the target value at clinic visits. TIQ was counted at first 200 consecutive patients, and the average value was 57.30 ± 147.20. *Conclusion*. The study presents the real-life data concerning the difficulties in hypertension treatment in patients with concomitant metabolic syndrome and/or type 2 diabetes mellitus. sBP was controlled at 32% patients only. The study results allow evaluating the effectivity of hypertension treatment as for drug choice, decrease of sBP and dBP associated with a certain drug, a drug combination, and therapeutic inertia in these patients.

## 1. Introduction

The aim of this study was to evaluate the effectivity of hypertension treatment as for drug choice, measure of decrease of systolic blood pressure (sBP) and diastolic blood pressure (dBP) associated with a certain drug, a drug combination, and therapeutic inertia in patients with metabolic syndrome and/or diabetes mellitus. It is well known from both clinical practice and literature how difficult it is to treat these patients with sBP and dBP to target values which are in this case a bit lower than the target values for hypertension patients without metabolic syndrome and/or diabetes mellitus. We have decided to follow the European Society of Hypertension and European Society of Cardiology 2013 guidelines (they were valid at the time of documenting the database), with systolic blood pressure goals for patients with diabetes mellitus <140 mmHg, diastolic blood pressure goals for patients with diabetes <85 mmHg, and blood pressure goals for patients with metabolic syndrome <140/90 mmHg [1, 2]. Thus, our unified BP target values used in this study were <140/90 mmHg, that is, a little less strict value than that could be expected with regard to the current SPRINT study results [3]. Our target values were based on the real-life data from our departments for outpatients, where BP values of 140/90 mmHg for diabetic (and often obese) patients could often be considered as a treatment success. This approach supports the 2016 Standards of the American Diabetes Association where the target values for diabetic patients are commonly determined as <140/90 mmHg. Lower BP targets, such as <130/80 mmHg, may be appropriate for certain individuals, such as younger patients, if they can be achieved without undue treatment burden [4].

Rather than the patients' proportion in the target values, more interesting to us were the questions about what measure of BP decrease would be associated with using certain drugs or what measure of BP decrease we can expect from this hypertension drug treatment. The same was followed for combination treatment.

To physicians, being effective in the hypertension treatment means not to remain passive at the regular patient visits and to adjust the drug choice and dosing to the actual BP values if they exceed the target BP. Therefore, the therapeutic inertia evaluation should be a part of the quality assessment in hypertension management [5–7].

## 2. Materials and Methods

We have analysed the database of 1,595 consecutive patients visiting our department of cardiology and internal medicine clinic for outpatients in 2005–2014; all the patients were in the secondary prevention area. The analysis included 13,990 visit records, and the average number of visits per patient was 8.5 ± 7.0.

**Figure 1:**
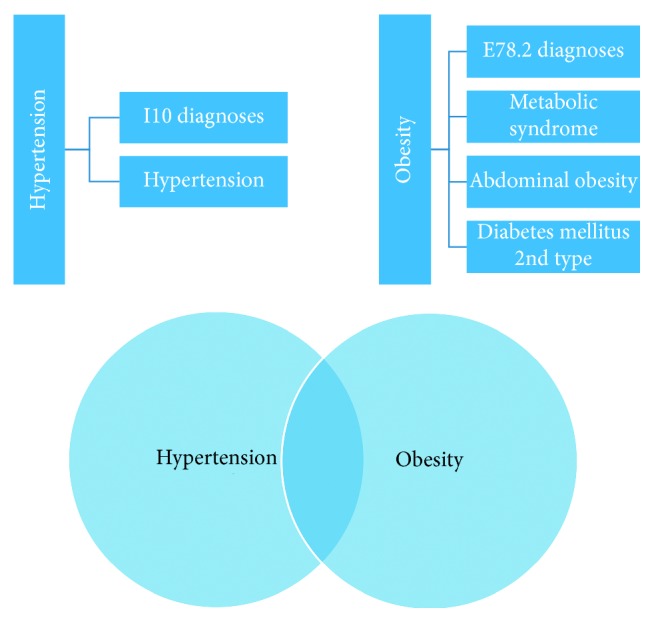
Patient selection from the database (interpenetration of 2 circles).

From the database, we have chosen a final number of patients who are as follows (Figure 1):

- Selected patients with I10 diagnoses.
- Selected patients with “hypertension” as a keyword.
- Previous two lists of patients have been merged into the first diagnostic circle.
- Selected patients with E78.2 diagnoses.
- Selected patients with “metabolic syndrome” as a keyword.
- Selected patients with “abdominal obesity” as a keyword.
- Selected patients with “diabetes mellitus” and “DM2” as a keyword.
- Previous four lists of patients have been merged into the second diagnostic circle.

We obtained a list of patients who had fulfilled the main intersection of both first and second circles. Subsequently, patients were filtered out so that each patient would be counted just once. We selected 636 patients meeting the requirement for both diagnostic circles. However, after removing those patients who had one medical visit only, no drug therapy, or whose blood pressure had not been not measured, we obtained a final database of 570 patients (304 males and 266 females, and the age average was 64 years).

### 2.1. The Procedure for Obtaining Data from the Database

For the final database of patients, following the alphabetical order, the data were assigned to the individual patient, that is, age, sex, systolic blood pressure values (SBP) and diastolic blood pressure values (DBP) at the beginning, during, and at the end of treatment, and antihypertensive drugs used for the hypertension treatment.

### 2.2. Calculation of Therapeutic Inertia (TIQ)

TIQ was calculated by determining the proportion of visits at which systolic blood pressure was above the target for eligible visits minus the proportion of these visits where the change was made only in antihypertensive treatment (AHT), either medication type or dose, over the number of total eligible visits, and the resultant value was multiplied by the mean of differences between the actual sBP and the target values at all the clinical visits.

TIQ = ((% visits with >TARGET − %Δ AHT/visits)/total number of visits) ∗ mean value of differences between actual sBP − TARGET sBP values during all visits.

**Figure 2:**
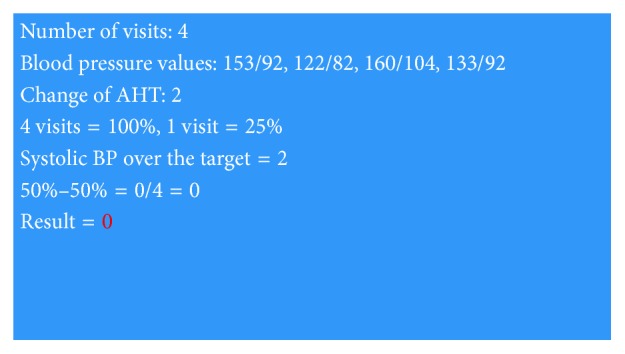
Example of the optimal value of inertia.

**Figure 3:**
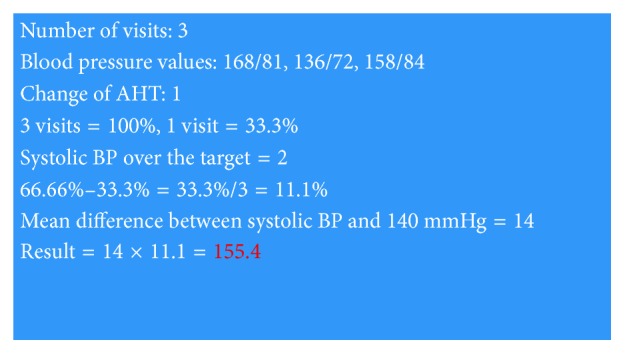
Example of the high value of inertia.

Theoretically, the best physician approach means to change the therapy at every visit during which the sBP exceeds the target value. Examples of optimal and high inertia calculations are in Figures 2 and 3.

## 3. Results

**Figure 4:**
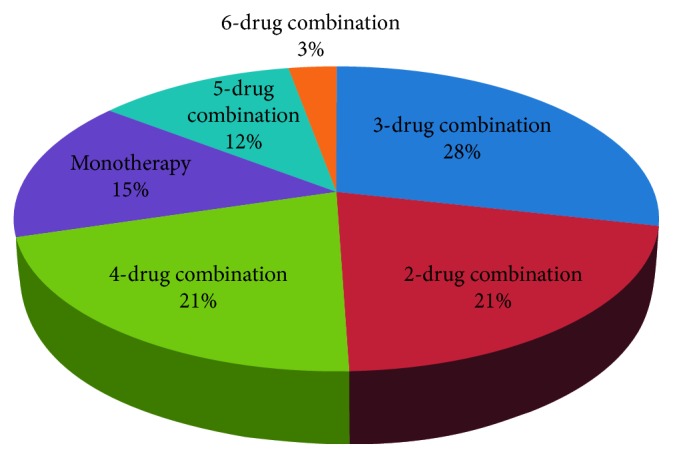
Monotherapy and combination therapy representation.

**Figure 5:**
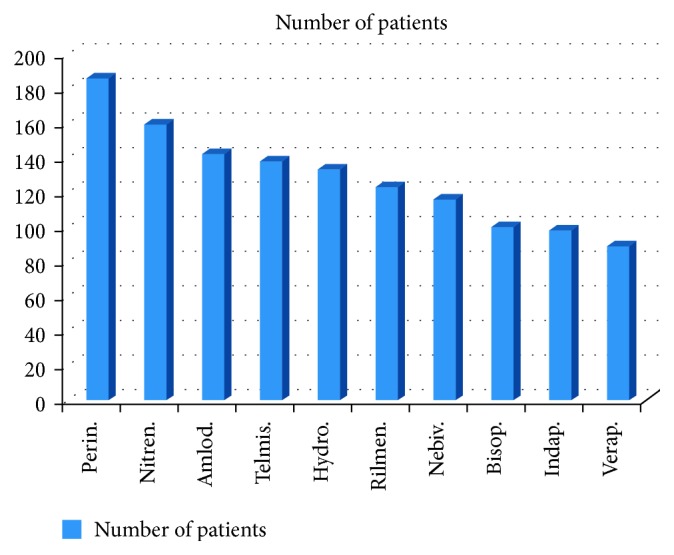
The number of patients treated using different drugs: “first league.” Perin. = perindopril, nitren. = nitrendipine, amlod. = amlodipine, telmis. = telmisartan, hydro. = hydrochlorothiazide, rilmen. = rilmenidine, nebiv. = nebivolol, bisop. = bisoprolol, indap. = indapamide, and verap. = verapamil.

**Figure 6:**
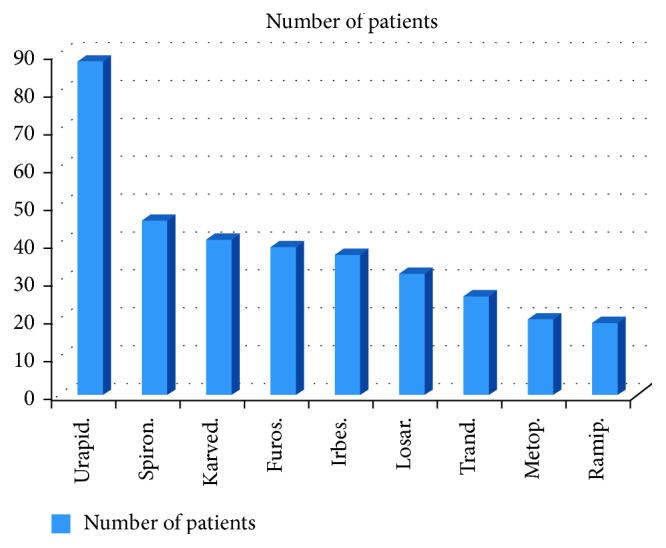
The number of patients treated using different drugs: “second league.” Urapid. = urapidil, spiron. = spironolactone, karved. = karvedilol, furos. = furosemide, irbes. = irbesartan, losar. = losartan, trand. = trandolapril, metop. = metoprolol, and ramip. = ramipril.

15% of patients were treated using hypertension monotherapy, 70% of patients were treated using 2- to 4-drug combination therapy, and 15% of patients were treated using 5- to 6-drug combination (Figure 4). The drugs used most frequently (Figure 5) were perindopril (perin), nitrendipine (nitre), amlodipine (amlo), telmisartan (telmi), hydrochlorothiazide (hydro), rilmenidine, and nebivolol (used in >100 patients, the so-called “first league”).  Figure 6 shows the drugs used less frequently (the “second league”). The most frequently used drugs and associated averaged changes of both sBP and dBP are presented in Table 1.

**Figure 7:**
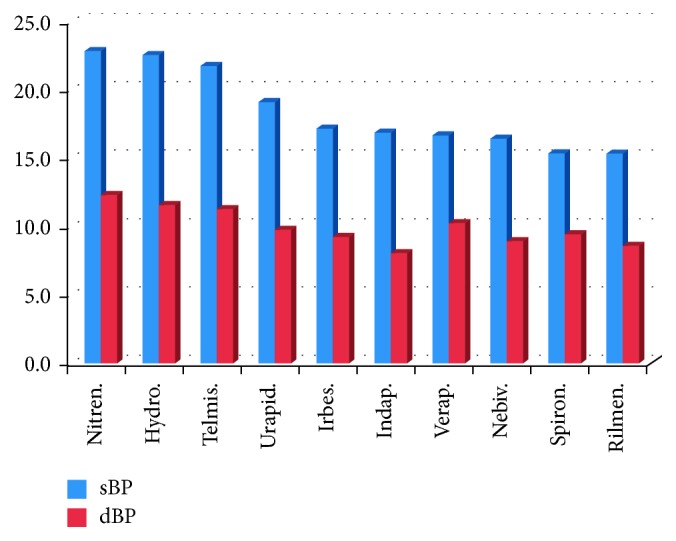
The most significant decrease of sBP and dBP associated with certain drugs (in mmHg). Nitren. = nitrendipine, hydro. = hydrochlorothiazide, telmis. = telmisartan, urapid. = urapidil, irbes. = irbesartan, indap. = indapamide, verap. = verapamil, nebiv. = nebivolol, spiron. = spironolactone, and rilmen. = rilmenidine.

**Figure 8:**
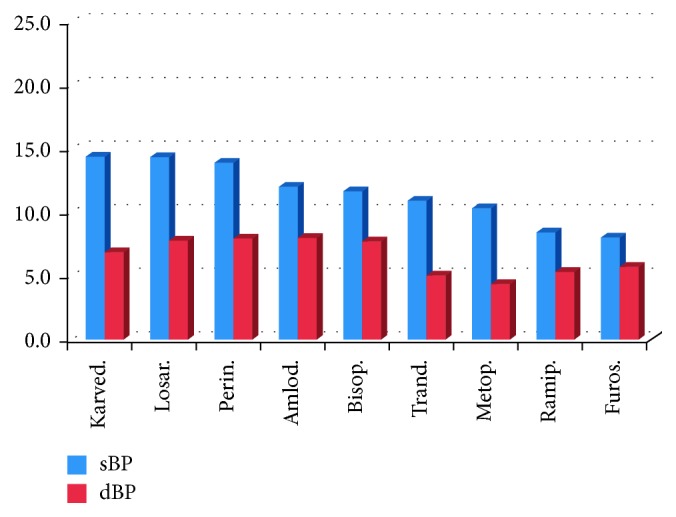
Less significant decrease of sBP and dBP associated with certain drugs (in mmHg). Karved. = karvedilol, losar. = losartan, perin. = perindopril, amlod. = amlodipine, bisop. = bisoprolol, trand. = trandolapril, metop. = metoprolol, ramip. = ramipril, and furos. = furosemide.

The most significant decrease of sBP (>19 mmHg) was associated with the treatment by nitre, hydro, telmi, and urapidil, and the most significant decrease of dBP (>10 mmHg) was associated with the treatment by nitre, hydro, telmi, and verapamil (Figures 7 and 8). As for the combination therapy, the effects of 2-drug combination and the associated averaged sBP and dBP are presented in Table 2. The most significant decrease of both sBP and dBP was associated with treatment using 3-drug combination (Table 3) of telmi + hydro + spironolactone (41 or 16 mmHg, resp.), telmi + hydro + nitre (34 or 15 mmHg, resp.), and telmi + hydro + urapidil (34 or 15 mmHg, resp.).

At the last visit, 281 out of 413 patients at the first visit had sBP >140 mmHg (68%); that is, sBP control was 32%. At the last visit, 76 patients out of 217 at the first visit had dBP >90 mmHg (35%); that is, dBP control was 65%.

TIQ was counted for the first 200 consecutive patients, and the average value was 57.30 ± 147.20.

## 4. Discussion

Hypertension jointly with abdominal obesity, combined dyslipidaemia, and insulin resistance constitutes a complex disease known as the metabolic syndrome. Hypertension and type 2 diabetes produce the so-called “lethal duo,” which increases the risk of cardiovascular disease 2- to 4-fold over both diseases themselves (heart attack, stroke, ischemic heart disease, and microvascular complications). There is a significant interference of antihypertensive drugs with glycaemic control, insulin resistance, lipid metabolism, and electrolyte balance. In the treatment of hypertension in patients with metabolic syndrome and/or diabetes mellitus, ACE inhibitors (or sartans) and calcium channel blockers are preferred for well-known nephroprotective, angioprotective, and metabolic effects. For these effects, these drugs are used even in the normotensive type 2 diabetic patients for albuminuria, retinopathy, and stroke prevention [8].

We have analysed the data of diabetics and metabolic syndrome patients together. Dividing the patients between diabetic and metabolic syndrome could lead to the decrease of the numbers of patients included in the group analysis and to the decrease of statistical power. Moreover, the margins between both groups are not sharp in every case, and patients suffering from diabetes usually belong to the metabolic syndrome group too. Analysis of the drug effectivity was not guided according to the class effect because there exist some differences between molecules inside every class group. Especially, their effectivities on high BP values were different as we can see in our results (different effect, for instance, between amlodipine and nitrendipine and between nebivolol and bisoprolol).

From the group of β blockers, in the view of the possible adverse reactions of β2 receptor blockade (inhibition of insulin release, masking the symptoms of hypoglycaemia), the use of β1 and selective vasodilators only (carvedilol and nebivolol) is recommended. Diuretics are particularly useful in the fixed combination of low-dose diuretic + ACE inhibitor or sartan. Calcium channel blockers are appropriate for this indication given their metabolic neutrality and excellent BP decrease efficiency. For patients with metabolic syndrome and type 2 diabetes mellitus, agonists of imidazoline receptors are suitable too, especially for their stimulation of insulin secretion, decrease of sympathetic hyperactivity, and inhibition of kidney sodium reabsorption [9, 10].

In our study, the drugs used most frequently were perindopril (perin), nitrendipine (nitre), amlodipine (amlo), telmisartan (telmi), hydrochlorothiazide (hydro), rilmenidine, and nebivolol, which corresponds to the recommendations mentioned above. More than 70% of patients were treated using 2- to 4-drug combinations, especially fixed combinations in the last years of the analysis. The most significant decrease of sBP (>19 mmHg) was associated with the treatment with nitre, hydro, telmi, and urapidil, and the most significant decrease of dBP (>10 mmHg) was associated with the treatment with nitre, hydro, telmi, and verapamil. The high efficiency of nitrendipine and telmisartan presented in the study was in concordance with our empirical, clinical experience. Nevertheless, we were surprised at the high position of hydrochlorothiazide treatment. However, it could be explained simply by the very common application of its fixed combination with telmisartan in our patient database.

In the case of the 2-drug combination, the most significant averaged sBP and dBP decrease was associated with the telmisartan-hydrochlorothiazide, nitrendipine-spironolactone, and irbesartan-hydrochlorothiazide combinations. The most significant decrease of both sBP and dBP was associated with treatment using the 3-drug combination of telmisartan + hydrochlorothiazide + spironolactone (41 and 16 mmHg, resp.), telmisartan + hydrochlorothiazide + nitrendipine (34 and 15 mmHg, resp.), and telmisartan + hydrochlorothiazide + urapidil (34 and 15 mmHg, resp.).

In spite of the really impressive effects of the drugs on BP values, the target values were reached for sBP in one-third of hypertensive patients and for dBP in two-thirds of the patients only. If we used the stricter target values (135/85 mmHg or less), then the hypertension control would be even lower. These data support the empirical experience from the clinical practice that hypertension pharmacotherapy in patients with concomitant metabolic syndrome and/or type 2 diabetes is difficult, where resistant hypertension is often expressed as a true form (in patients with obesity, sleep apnoea syndrome, and autonomic neuropathy) or as a false form (nonadherence).

Therapeutic inertia is defined as the provider's failure to change the therapy when treatment goals are unmet and contributes to higher prevalence of uncontrolled hypertension. Regular assessment of the inertia score leads to decreased score in time and refers to the accuracy of the therapy. Our score of 57.3 is comparable to the results of some other studies [4–6]. The higher SD value could reflect the database's heterogeneity and a higher variability of the BP values which is typical especially of diabetic patients with autonomic neuropathy.

### 4.1. Limitations

Patient selection in the database was not carried out in accordance with the Adult Treatment Panel or American Diabetes Association criteria, but using keywords. Data on 24 h ambulatory blood pressure monitoring are lacking; in our country, this method is not applied in all the patients treated for hypertension, and the indication is restricted for the special cases. Effectivity according to the dosage evaluation was not done. It could lead to the decrease of the numbers of patients included in the group analysis and to the decrease of statistical power. Dosage at every patient was adjusted according to the actual BP values, and dosage was currently changed during the follow-up. So, it was impossible to choose only one dose for analysis. Home BP values referred by patients were also taken into account during the dosing process. Mutual interactions of many drugs used in hypertension therapy, often more than 3-drug combinations, were not taken into consideration, and adjustment for another drug's effects, weight, salt reduction, and so on was not performed. The whole database was recorded by one experienced clinical cardiologist only, which means that personal preferences could influence the drug choice. However, no conflicts of interest were present to disclose, and principal effort was only to reach optimal BP control based on more than 40 years of clinical experience and continual medical education.

## 5. Conclusion

The study presents the real-life data concerning the difficulties of hypertension treatment in patients with concomitant metabolic syndrome and/or type 2 diabetes mellitus. sBP was controlled in 32% of patients only. The study results allow evaluating the effectivity of hypertension treatment as for the drug choice, decrease of sBP and dBP associated with a certain drug, a drug combination, and therapeutic inertia in patients with metabolic syndrome and/or diabetes mellitus. The score of therapeutic inertia evaluation may contribute to the improvement of hypertension control and should be a part of the quality assessment in hypertension management.

## Figures and Tables

**Table 1:** Most frequently used drugs and associated BP changes.

	Number of patients	Start		Finish		Average decrease of sBP	Average decrease of dBP	SD sBP	SD dBP
		Average sBP	Average dBP	Average sBP	Average dBP				
Perindopril	186	154.1	88.5	140.2	80.6	13.9	7.9	22.7	13.7
Nitrendipine	159	165.1	89.7	142.3	77.5	22.8	12.2	29.0	15.4
Amlodipine	142	153.0	88.0	141.0	80.0	12.0	8.0	24.0	14.0
Telmisartan	138	161.8	88.8	140.2	77.6	21.7	11.2	28.9	15.1
Hydrochlorothiazide	133	161.8	89.0	139.2	77.5	22.5	11.5	29.7	15.9
Rilmenidine	123	159.1	90.3	143.9	81.8	15.2	8.5	28.8	15.8
Nebivolol	116	158.3	89.6	141.9	80.8	16.4	8.9	24.5	13.9
Bisoprolol	100	150.0	84.5	138.4	76.8	11.6	7.7	23.1	15.7
Indapamide	98	156.2	88.4	139.4	80.4	16.8	8.0	21.4	12.9
Verapamil	89	157.7	89.4	141.1	79.2	16.6	10.2	24.2	13.6

**Table 2:** Combination of two drugs: effect on BP values.

	Number of patients	Start		End		Average decrease of sBP	Average decrease of dBP
		Average sBP	Average dBP	Average sBP	Average dBP		
Telmisartan	83	165	90	138	76	27	14
Hydrochlorothiazide							
Irbesartan	13	161	89	140	77	21	12
Hydrochlorothiazide							
Perindopril	87	157	91	142	81	15	9
Amlodipine							
Perindopril	36	156	87	137	78	19	9
Nitrendipine							
Nitrendipine	12	181	89	157	82	24	7
Spironolactone							

**Table 3:** Combination of three drugs: effect on BP values.

	Number of patients	Start		End		Average decrease of sBP	Average decrease of dBP
		Average sBP	Average dBP	Average sBP	Average dBP		
Telmisartan	21	175	90	141	75	34	15
Hydrochlorothiazide							
Urapidil							
Hydrochlorothiazide	3	155	81	145	69	10	12
Urapidil							
Irbesartan							
Nitrendipine	44	170	90	136	74	34	15
Telmisartan							
Hydrochlorothiazide							
Spironolactone	9	179	86	139	70	41	16
Telmisartan							
Hydrochlorothiazide							
